# Electrical Resistivity Measurements of Reinforced Concrete Slabs with Delamination Defects

**DOI:** 10.3390/s20247113

**Published:** 2020-12-11

**Authors:** Kevin Paolo V. Robles, Dong-Won Kim, Jurng-Jae Yee, Jin-Wook Lee, Seong-Hoon Kee

**Affiliations:** 1 Department of ICT integrated Ocean Smart Cities Engineering, Dong-A University, Busan 225, Korea; kpvrobles@donga.ac.kr (K.P.V.R.); kdw9405@donga.ac.kr (D.-W.K.); jjyee@dau.ac.kr (J.-J.Y.); 2 Principal Researcher, Advanced Railroad Civil Engineering Division, Korea Railroad Research Institute, 176 Choldobangmulgwan-ro, Uiwang-si, Gyeonggi-do 176, Korea

**Keywords:** electrical resistivity, concrete, delamination defects, non-destructive evaluation

## Abstract

The main objectives of this research are to evaluate the effects of delamination defects on the measurement of electrical resistivity of reinforced concrete slabs through analytical and experimental studies in the laboratory, and to propose a practical guide for electrical resistivity measurements on concrete with delamination defects. First, a 3D finite element model was developed to simulate the variation of electric potential field in concrete over delamination defects with various depths and lateral sizes. Second, for experimental studies, two reinforced concrete slab specimens (1500 mm (width) by 1500 mm (length) by 300 mm (thickness)) with artificial delamination defects of various dimensions and depths were fabricated. Third, the electrical resistivity of concrete over delamination defects in the numerical simulation models and the two concrete slab specimens were evaluated by using a 4-point Wenner probe in accordance with AASHTO (American Association of State Highway and Transportation Office) T-358. It was demonstrated from analytical and experimental studies in this study that shallow (50 mm depth) and deep (250 mm depth) delamination defects resulted in higher and lower electrical resistivity (ER) values, respectively, as compared to measurements performed on solid concrete locations. Furthermore, the increase in size of shallow defects resulted in an increase in concrete resistivity, whereas the increase in sizes of deep delamination defects yielded opposite results. In addition, measurements done directly above the steel reinforcements significantly lowered ER values. Lastly, it was observed from experimental studies that the effect of delamination defects on the values of electrical resistivity decreases as the saturation level of concrete increases.

## 1. Introduction

Concrete, a key component in buildings and infrastructures [1], is one of the most widely used building and construction materials due to its predominant advantages, such as excellent plasticity, satisfactory waterproofness, durability in harsh environments, and cost-effectiveness as compared to other construction materials [1,2]. It is twice as abundant as all other building materials in the world [3]. This results in abundant production of cement in the world market, which causes 6% of total carbon dioxide emissions [4]. According to Yekkalar et al., (2013), as long as the increase of the durability of concrete structures is prioritized, this would reduce the consumption of raw materials and natural resources, and ultimately decrease construction waste [5]. That is why a substantial budget from many countries and states has been expended for the repair, improvement, and maintenance of such structures [6]. In the United States alone, it is estimated that 1.6 trillion dollars were used for infrastructure rehabilitation from 2011 to 2015 [7]. As per Pacheco-Torgal (2017), the “Law of Fives” is applicable for the service life of concrete structures. Every dollar ($1) that is spent for design and construction equates to $5 when the damage begins; $25 dollars at the start of the deterioration; and $125 for extensive damages [8]. 

Corrosion of reinforcing bars (rebars) in concrete is known to be a major source of deterioration of concrete in combination with other mechanism such as freezing and thawing cycles and carbonation [6,9,10]. Rebars in concrete with strong alkalinity (i.e., pH 12~13) corrode very slowly due to the presence of passive film with an insoluble substance on the surface of the rebars. However, the passive film is unstable when the pH of the concrete is below 9 or when the chloride ion concentration in the concrete is above a certain level. Being exposed to the corrosive environment due to sufficient moisture and oxygen in the pores of the concrete, rebars tend to corrode because of equilibrium with the environment. It is important to evaluate the corrosive environment and the corrosion activity of rebars, which is essential to better understand the current condition of concrete and, if needed, to decide appropriate maintenance actions [11].

The electrical resistivity (ER) method is one of the most suitable and easiest non-destructive evaluation (NDE) method to characterize concrete’s susceptibility to corrosion by evaluating its corrosive environment. Previous researchers have demonstrated that ER values of concrete can be correlated with its durability parameters such as corrosion rate [12,13,14,15,16], chloride diffusivity [17,18,19,20,21], and compressive strength [22,23,24,25,26]. It has been established in prior studies that the concrete resistivity is inversely proportional to corrosion, i.e., a decrease in ER results in an increase in corrosion rate, and vice versa. Similarly, a higher chloride diffusivity also results from decreasing ER measurements. A decrease in ER measurements means a higher chloride diffusivity and chloride penetration. On the other hand, ER varies linearly with the compressive strength of concrete, and is also correlated with porosity of concrete. A higher compressive strength (or lower porosity) will result in a rise in concrete ER values. Therefore, ER measurements can also help in identifying regions of the reinforced concrete elements susceptible to chloride penetration. In addition, ER surveys can be used to evaluate corrosion activity of concrete with another corrosion-detection techniques, such as half-cell potential that evaluates the probability of corrosion. 

**Figure 1:**
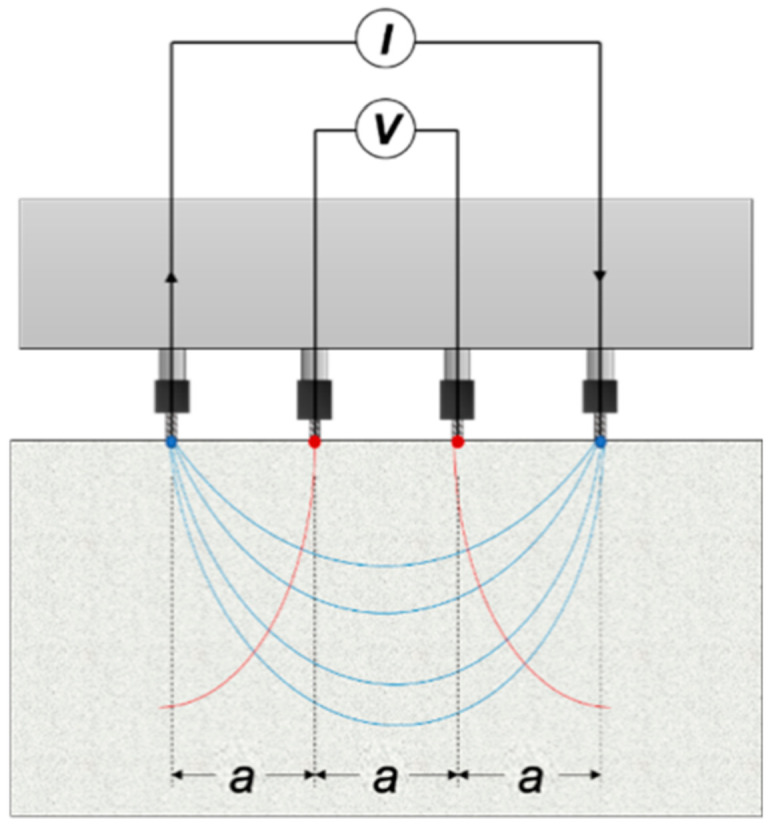
Wenner probe configuration, with “a” as the distance between the electrodes.

In practice, the voltage and current are measured at the surface of the object under investigation. The most common electrode layout in civil engineering applications is the Wenner setup, which was invented in 1905 for geology [27]. In a Wenner probe configuration, four electrodes are aligned at an equal distance with each other (see Figure 1). The external current is imposed at the two exterior electrodes and the electrical potential difference is measured through the two internal electrodes. ER is then calculated according to the following equation.

(1) $$\rho =kR=k\left(\frac{V}{I}\right)$$

where ρ is resistivity, *V* is voltage, and *I* is current. The geometrical constant k is dependent on the size and shape of the specimens and electrode spacing. In the laboratory, the ER equipment is usually used for cylindrical and prismatic specimens while on the field, it is used in concrete bridge decks, slabs, beams, and columns. For field applications, some researchers successfully visualize the corrosive environment of concrete in actual structures using a Wenner probe setup. Recently, a robotics-assisted bridge inspection tool (RABIT) was developed and deployed on the actual bridges [28]. The RABIT combines the capability of multiple NDE techniques, which includes four Wenner probes to automatically measure ER of concrete bridge decks. It has been demonstrated that the RABIT is effective for optimizing and speeding-up the monitoring and comprehensive evaluation of bridge decks.

The data processing of ER method is simple and easily reduces to plotting the raw data. However, the interpretation of ER data in concrete is more challenging. One of the reasons is that the ER value of concrete is sensitive to its material properties and various environmental and external factors, which include water/cement ratio [29,30], age of concrete [31,32], moisture content and degree of saturation [33], specimen geometry [34], temperature [35,36], electrode spacing [37], presence of rebars [38,39], cracks [40,41], and delamination defects [40].

Meanwhile, delamination defects are usually found beneath the surface of the concrete located at the upper layer, between two layers, or below the steel reinforcements [42]. It is considered as a subsurface fracture plane present in the concrete due to the corrosion of the embedded rebars. The presence of delamination defects could change the boundary conditions of the electrical field, which causes variations of geometrical constant during ER measurements. Therefore, validating the presence and effect of delamination in concrete to ER measurements is important to be able to establish an appropriate decision for its monitoring and maintenance [43].

However, there is only limited prior research regarding the effect of delamination defects on the ER measurements. For the influence of delamination defects to ER measurements, researchers only indicated that delamination causes difference in resistivity values. Chouteau and Beaulieu (2002), in their numerical investigation, found out that ER values over delamination defects in concrete were different from those values measured over sound concrete [40]. In the experimental study by Lataste (2003), the average resistivity values measured at sound concrete zones is around 800 Ω-m, while the average measurements on delaminated zones is around 1700 Ω-m and the maximum resistivity reaches 3000 Ω-m [41]. Morales (2014) conducted an experimental setup creating delaminated zones by putting plastic sheets on top of the rebar mesh with different concrete covers and saturation conditions. It was concluded that the variations in relative ER (ratio of apparent ER measured from delaminated zones over the apparent ER measured over solid concrete) are largest at the smallest concrete cover thickness [44]. In summary, no systematic approach has been developed to determine the interference of delamination defects in concrete with the electrical resistivity.

The primary purposes of this research are to evaluate the effects of delamination defects in reinforced concrete slabs on the ER measurements and to propose a practical guide for ER measurements in concrete with delamination defects. For these purposes, four main tasks were performed in this study. First, a 3D finite element model was developed to simulate the variation of electric potential field in concrete over delamination defects with various depths and lateral sizes. Second, for experimental studies, two reinforced concrete slab specimens (1500 mm (width) by 1500 mm (length) by 300 mm (thickness)) with artificial delamination defects of various dimensions and depth were fabricated. Third, the electrical resistivity of concrete over delamination defects in the simulated concrete slabs and the two concrete slab specimens were measured by using a 4-point Wenner probe in accordance with AASHTO T 259. Lastly, the effects of delamination defects on ER measurements were systematically investigated and discussed based on collected data from a series of numerical simulations and experiments in this study. 

## 2. Numerical Simulation

### Model Description

**Figure 2:**
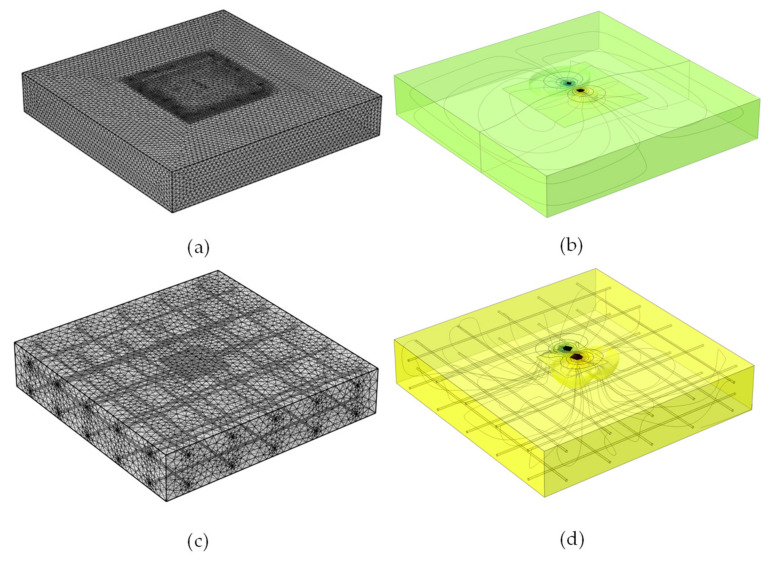
3D finite element models for simulation of electrical field over a delamination defect in concrete: (**a**,**c**) finite element models for a plain- and a reinforced concrete slab, respectively; (**b**,**d**) electric potential field over a delamination defect in a plain- and a reinforced concrete slab, respectively.

A 3D finite element model was developed to simulate the interference of electric potential field with a delamination defect in concrete using a commercially available numerical simulation tool based on finite element method, AC/DC module in the COMSOL Multiphysics v5.5, as shown in Figure 2. A plain concrete slab (width = 1500 mm, length = 1500 mm, thickness = 300 mm) was simulated and studied with approximately four million extremely fine tetrahedral mesh elements with a total mesh volume of 0.675 m^3^ (see Figure 2a). Four electrodes with distance of 38 mm, similar to that of the commercial Wenner probe device, was placed at the center of the surface of the slab (see Figure 2b). In this study, a reinforced concrete slab was also simulated to investigate the effect of rebar meshes embedded in concrete on ER measurements (see Figure 2c,d). The reinforced concrete slab model had the same dimensions and comparable mesh density compared to the plain concrete slab model. 

This numerical simulation calculated the electric potential using the classical Poisson’s equation derived using Gauss law and equation of continuity,

(2) $$-\nabla \cdot \left(\sigma \nabla V-J^{e}\right)=Q_{j}$$

where σ is the electrical conductivity (inverse of ER), $J^{e}$ is the externally generated electric current, and $Q_{j}$ is the current source. 

Using an input true resistivity of 100 kΩ-cm and an external current of 200 μA placed at the two external electrodes, the electric potential difference (in V) were measured using two boundary probes placed at the location of the two internal electrodes. For comparison to the experimental data, the simulated potential difference was used to compute for the apparent ER using Equation (1).

In this study, a series of numerical simulations was conducted to investigate the effect of geometric properties (depth and size) of artificial delamination defects in concrete and the presence of rebars on the effect of delamination defects. For the effect of the delamination depth, a model with a 300 mm square delamination was simulated at varying depths, starting from 25 mm to 275 mm deep with intervals of 5 mm. For the effect of delamination size, artificial square delamination defects with dimensions ranging from 25 mm to 1000 mm were studied, at a constant depth of 50 mm (shallow) and 250 mm (deep).

## 3. Experimental Study

### 3.1. Preparation of Concrete Slab Specimens

**Figure 3:**
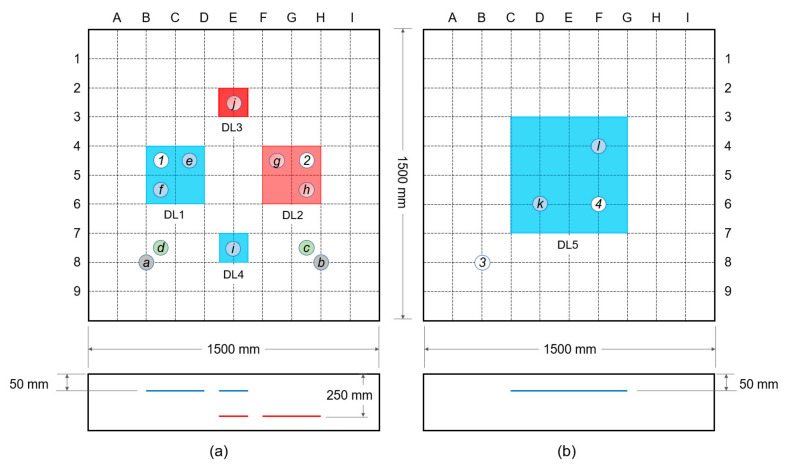
Reinforced concrete slabs with artificial delamination defects with different sizes and depths: (**a**,**b**) concrete slab specimens 1 and 2, respectively.

**Figure 4:**
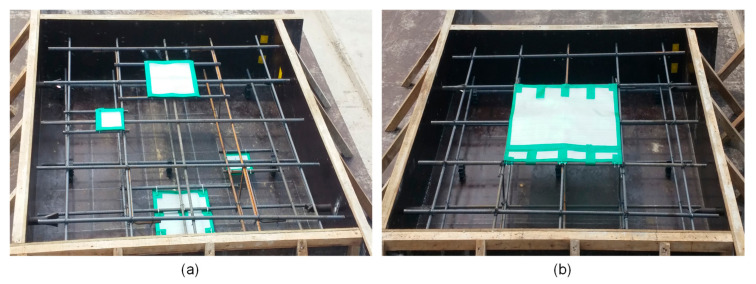
Actual orientation of the delamination defects installed to the reinforcing steel bars: (**a**) shallow and deep delamination defects in the concrete slab specimen 1, (**b**) a shallow delamination defect in the concrete slab specimen 2.

Two 1500 mm (length) by 1500 mm (width) by 300 mm (thickness) reinforced concrete slabs were fabricated at Dong-A University as part of a research project for the condition assessment and evaluation of old and deteriorated subway concrete tracks in the cities in South Korea. The concrete specimens were both composed of Type I Portland cement (440 kg/m^3^), river sand (701 kg/m^3^), crushed coarse aggregate (1049 kg/m^3^), and water (165 kg/m^3^), with a total weighted density of 2355 kg/m^3^ and a water/binder ratio of approximately 0.38. The mixture was designed to 28-day compressive strength of 35 MPa. Two layers of uncoated reinforcing steel bars with a diameter of 13 mm were placed in two-way orientation with center-to-center spacing of 300 mm placed at 50 mm (top layer) and 250 mm (bottom layer) depths. Artificial delamination defects using double-layered thin film with a thickness of 50 μm were incorporated in the manufactured concrete slabs and were placed at different depths with different sizes as shown in Figure 3 and Figure 4. Shallow delamination defects (blue) were placed at 50 mm depth directly above the rebars, whereas deep delamination defects (red) were placed at 250 mm depth and directly below the rebars. The concrete slab specimens were kept in air dried condition with a temperature of 20 ± 3 °C in the laboratory.

### 3.2. Electrical Resistivity Measurements

#### 3.2.1. Test Setup

**Figure 5:**
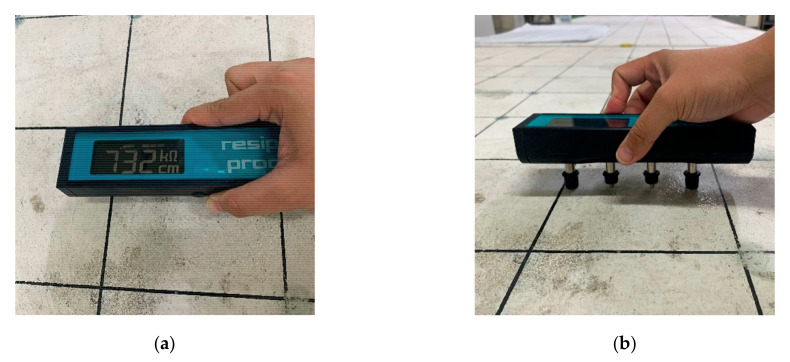
Measurement of ER on a concrete slab surface using a Wenner probe device: (**a**) top view and (**b**) side view of the testing device.

Electrical resistivity (ER) of concrete was measured on the surface of the two concrete slabs by using a commercially available device (Resipod Proceq), which is based on the 4-point Wenner Probe principle (see Figure 5). The device has a probe spacing of 38 mm (1.5 inches) which conforms to the standard specifications of AASHTO T-358 for the surface resistivity test method. According to the device’s manual, an input current ranging from 10 μA to 200 μA is driven to the concrete, depending on the specimen’s contact resistance [45]. The output values are displayed in kΩ-cm, the unit of measurement for ER.

#### 3.2.2. ER Measurements with Various Concrete Saturation Conditions

It is important to examine the effect of saturation conditions on ER measurements on concrete slabs since ER is strongly dependent on water content in concrete. ER values of dried concrete is extremely high that often exceeds the capacity of the measurement device. Accordingly, a number of researchers recommended wetting the surface by using a sponge or spraying a little amount of water before making a measurement [46,47,48,49,50]. However, there has been no standard guide for determining the amount of water (or standard saturation condition) for ER measurement. 

In this study, ER of concrete was measured over four locations on the surface of concrete with various saturation conditions to investigate the effect of saturation conditions on ER measurements. Four points were used to conduct this test (see Figure 3a,b): point ① at DL1 (a large shallow delamination at specimen 1); point ② at DL2 (a large deep delamination at specimen 1); point ③ for solid concrete at specimen 2; and point ④ at DL2 (a shallow delamination at specimen 2). The probes were placed parallel to the horizontal axis and the resistivity were measured every minute. Instantaneous saturation of the concrete surface was measured for a total duration of 50 min. 

In addition, ER of concrete was measured on five concrete cylinders (100 mm diameter and 200 mm height) with various saturation conditions of concrete to investigate the relationship between the degree of saturation and ER of concrete. The concrete cylinders were made with same concrete composition that were used for fabrication of the two concrete slab specimens. The concrete cylinders were immersed in water for 10 days, starting from oven dry condition, reaching a fully water saturated condition. The weight of concrete cylinders was measured before immersion and was continuously measured with the ER for every 20 min for the first one hour, every 30 min for the next nine hours, and every 24 h for a total of 10 days. In measuring the surface resistivity of the concrete cylinders, the Wenner probe device was placed at every 90° parallel to the height of the concrete cylinder, for two complete rotations in accordance with AASHTO TP95-11. The average concrete resistivity was computed from the average measurements of the five samples, which were computed individually by also averaging a total of eight measurement from four different locations. The degree of saturation (DS) was calculated by dividing the water content measured on a specific time over the maximum water content measured for the whole ten-day immersion. Mathematically, the equation for degree of saturation is:

(3) $$D.S.\;=\;\frac{m_{i}-m_{OD}}{m_{SSD}-m_{OD}}$$

where $m_{i}$ is the instantaneous mass of the specimen at specific time of saturation, $m_{OD}$ is the mass of the oven-dry (OD) concrete cylinders, and $m_{SSD}$ is the mass of the saturated-surface-dry (SSD) concrete cylinders.

#### 3.2.3. ER Measurements over Various Delamination Defects

The electrical resistivity (ER) values of the two specimens were determined at different points presented as circled letters in Figure 3. Measurements in the concrete slab specimen 1 were made at several selected testing locations: points ⓐ and ⓑ over solid concrete, points ⓒ and ⓓ over solid concrete near a rebar, points ⓔ and ⓕ over a large shallow delamination defect (DL1), points ⓖ and ⓗ for a large deep delamination defect (DL2) and points ⓘ and ⓙ for small shallow (DL3) and deep delamination (DL4) defects, respectively. For specimen 2, measurements were made at two testing points ⓚ and ⓛ over a large delamination defect (DL5). In order to remove the contact resistance between the concrete and the electrodes, but to assure that the concrete be maintained in air dry condition, the surface of the location being tested was sprayed with water twice, and the water was spread uniformly on the surface through a sponge. Measurements were done during the first two minutes of surface saturation to clearly identify the differences in ER measurements. Thirty measurements were repeated at each individual test location with a single probe configuration. The locations of the rebars were determined using a portable GPR system (StructureScan Mini XT produced by Geophysical Survey Systems Inc. (GSSI), Nashua, NH, USA).

**Figure 6:**
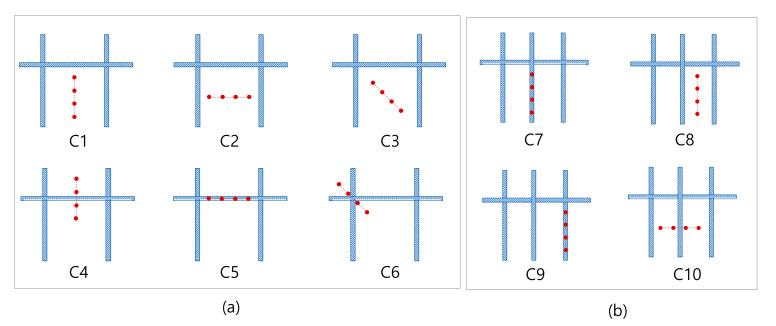
Wenner probe configuration: (**a**,**b**) for large and small delamination defects in concrete slab specimen 1.

At every point indicated, six probe configurations, shown in Figure 6, are established to check if the presence of rebars has a significant effect to the ER measurements. Several research papers established that the presence of rebars results in an alteration to the ER of concrete [37,39,51,52,53,54]. In relation to this, a modified configuration from the thesis paper by Salehi et al., (2016) was followed. With reference to the A-I axis, electrode configurations for large delamination defects of specimens were placed in vertical orientation (C1), horizontal orientation (C2), diagonal orientation (C3), perpendicular to a rebar (C4), directly above the rebar (C5), and diagonal to a rebar mesh (C6). For small delamination defects, the Wenner probe was placed in vertical configurations at three different locations (C7, C8, and C9), and a horizontal configuration (C10). Only three configurations were made for the solid concrete and points considered in the shallow delamination in specimen 2 since it was far from any reinforcement bar: horizontal, vertical, and diagonal configurations. Accordingly, the edges of the slab were disregarded in considering the measurement points to avoid the effect of the specimen geometry in the values of electrical resistivity. According to Sengul and Gjorv (2008), there will be an overestimation of ER when measurements are made closer to the edges of the concrete specimen [55].

## 4. Results and Discussion

### 4.1. Experimental Variability of Electrical Resistivity Measurements

Experimental variability of the measured electrical resistivity (ER) is of interest when investigating the consistency and reliability of the test methods. In this study, thirty measurements were performed at each individual test location, with a single probe configuration, to investigate experimental variability of the measured ER values. The coefficient of variation (COV, the standard deviation, σ, divided by the mean value, μ, of a set of specimen) was used as a means of evaluating the experimental variability of ER measurements on the surface of concrete slab specimens. Table 1 and Table 2 summarizes the statistical parameters (μ and COV) of the ER values measured over solid concrete and shallow and deep delamination defects, with different probe configurations following the method described in Section 3.2.3. According to AASHTO TP 358-15 [56], the single test single-operator COV for laboratory evaluation of concrete samples is 6.3%. It can be seen in Table 1 that the COV of all measurements are either lower or near the standard COV set. This establishes that the data gathered in this experiment for solid concrete and delaminated zones are controlled and consistent.

It was also observed that the distribution of ER values follows the normal distribution after the Kolmogorov–Smirnov (K-S) test. Table 3 and Table 4 summarizes the K–S statistics (D), which shows all D values (except the point ⓗ with the configuration C5) are lower than the critical D values (i.e., 0.2417 for sample size, *N* = 30). It can be interpreted that the measured ER data can be represented by two statistical parameters (i.e., mean value and standard deviation). 

### 4.2. Effect of the Depth of Delamination Defects

**Figure 7:**
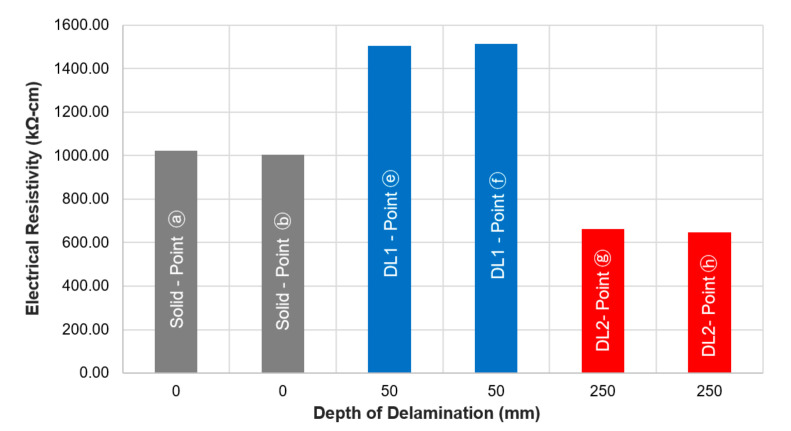
Average electrical resistivity values of concrete measured at the surface of the concrete slab specimen 1 over solid concrete and shallow and deep delamination defects, with probe configuration C1.

Figure 7 shows the average ER values measured at the surface of the concrete slab specimen 1 over solid concrete (ⓐ and ⓑ), shallow (ⓔ and ⓕ, with a depth of 50 mm) and deep delamination defects (ⓖ and ⓗ, with a depth of 250 mm), with a uniform probe configuration (C1). The delamination defects shown in this figure have the same lateral dimensions of 300 mm by 300 mm. The ER measurements were made about 1–2 min after spreading water by sponge on the surface of the concrete specimen. It was observed that shallow delamination defects result in higher ER values as compared to measurements made at solid concrete (ⓐ and ⓑ), whereas lower ER values were determined at parts with deep delamination defects. The average measured ER at the shallow delaminated regions (ⓔ and ⓕ) was 1508.4 kΩ-cm, which is about 50% greater than the average ER value at solid concrete, 1005.4 kΩ-cm. In contrast, the average ER value measured at the deep delaminated regions was 648.1 kΩ-cm, about 35% lower compared to the values at solid concrete.

**Figure 8:**
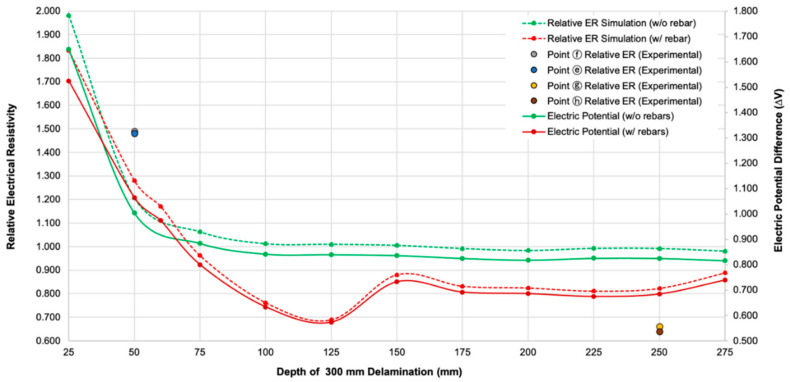
Variation of the electric potential difference and relative electrical resistivity (ER) of concrete slab with depth of delamination defects measured from numerical simulation.

**Figure 9:**
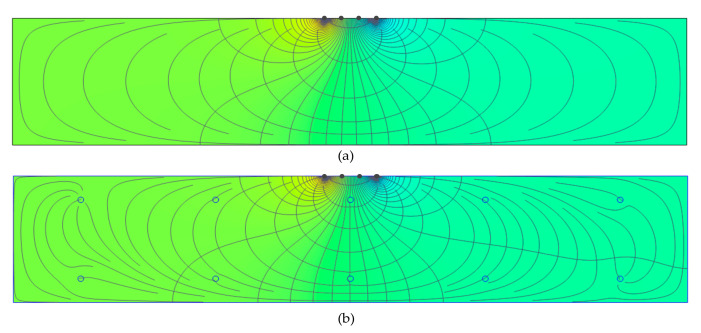
Electric potential field and equipotential distribution in the middle section concrete slab models: (**a**) plain concrete model and (**b**) reinforced concrete model.

Figure 8 shows the variation of the electric potential difference (∆V) and relative resistivity (ratio of apparent ER over a delamination defect (300 mm width by 300 mm length) in concrete over apparent ER of solid concrete slab) with various depth of a delamination defect in concrete obtained from numerical simulations in this study. Following Equation (1), ρ has a linear relationship with ∆V which explains why both data have the same trend (see Figure 8). The relative ER values evaluated from the plain concrete and reinforced concrete models (see Figure 2) are presented as green and red circles with dashed lines, respectively. For comparison, relative ER data measured from the concrete slab specimen 1, shown in Figure 7, are also presented as solid circle in Figure 8. Overall, the relative ER based on numerical simulations decreases with increasing depth of a delamination defect. The relative ER from the plain concrete converges to approximately 1.0, which means the effect of delamination defects becomes ignorable when the depth of a delamination defect is sufficiently deep. However, this observation is different from those obtained from actual concrete slab specimen in the laboratory. Relative electrical resistivity measured over the shallow delamination defects (depth of 50 mm) were 1.48 and 1.49, for points ⓔ and ⓕ as shown in the Figure 9 (blue and gray plot), respectively, whereas according to the experimental data for deep delamination, the relative resistivity for points ⓖ and ⓗ, are 0.66 and 0.64 (brown and yellow plot). Other influential factors (e.g., the degree of saturation, heterogeneity of concrete, and the presence of reinforcing bars) could affect the ER values in actual concrete slab specimens. The simulation results from the reinforced concrete model in Figure 9, validate the experimental data, showing that the presence of rebars could cause the increase in relative ER over the shallow delamination (50 mm). Furthermore, it could result in significant decreases in ER values over the deep delamination defects. It is observed in Figure 9 that the presence of rebar caused a disruption in the current flux and equipotential lines distribution throughout the concrete slab causing variation in the electric potential difference. However, since the orientation of steel reinforcement is unique and varying in different reinforced concrete structures, a more detailed and in-depth study of the effect of such (and other environmental factors) to ER values is still needed.

### 4.3. Effect of the Width of Delamination Defects

Table 5 shows the ER values measured at different sizes of delamination defects in the concrete slab specimens in this study. Comparing values using probe configuration C1, it can be inferred in the table that for measurements done at shallow delaminated zones (DL1, DL3, and DL5), the ER value increases as the size of the artificial delamination increases. For the 150 mm shallow delamination defect (DL4), a value of 1362.0 kΩ-cm is measured, a larger ER of 1504.4 kΩ-cm for the 300 mm delamination defects (DL1), and an overflowing (OF) value for the largest (600 mm) delamination defect (DL5).

**Figure 10:**
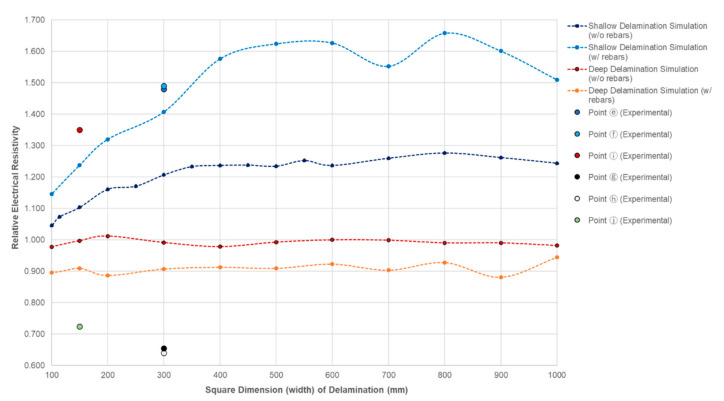
Variation of the relative electrical resistivity of concrete slab with varying lateral sizes of delamination defects.

The experimental result for the shallow delamination in Table 5 was compared with the results obtained from the numerical simulation using both the plain concrete and reinforced concrete model. Figure 10 shows that the increase of the dimension of the square artificial delamination in the plain concrete results in the gradual increase in the simulated relative ER of concrete slab. It shows that relative ER increase to as much as 1.28 for the 800 mm square dimension. For comparison, the graph also presents the plot of the experimental data for the 150 mm (yellow circle) and 300 mm (blue and gray circles) delamination defects that lies above the simulated graph. It can be observed in Figure 10 that a simulated reinforced concrete model shows a larger increase in relative ER, as compared to plain concrete. Steel, being a more conductive material than concrete, will result to variation in ER measurements. Nonetheless, both experimental and simulated data follow the same trend.

For deep delamination defects, on the other hand, the numerical simulation of the plain concrete and reinforced concrete models show a difference in results. The relative ER of the reinforced concrete is lower as compared to the plain concrete. Both the simulation models give a steady trend at increasing dimensions of delamination defects. It can be deduced from the numerical simulation of the plain concrete that the size of deep delamination defects does not affect the measurement of the ER, having a relative ER of nearly 1.0. Moreover, this, and the reinforced concrete simulation, prove that the presence of rebar caused the decrease in relative ER (approximately 0.9) for the reinforced concrete model. These results also validate measurements gathered for deep delamination defects (DL2, and DL4), having experimental relative ER of 0.66 and 0.64, respectively. It should be noted that as explained in Section 4.2, in an actual scenario, aside from the steel reinforcements, other environmental factors contribute to the decrease of ER. Moreover, for both delamination defects, additional rebars are installed to fully support the placement of the artificial delamination before concrete pouring, which could be attributed to the differences in the relative ER values from experiments and numerical simulations in this study.

### 4.4. Effect of Configuration of Wenner Probe Device

It is indicated in Table 1 that among the six device configurations studied in this experiment, probe configuration C5, placed directly above a rebar, has the lowest set of ER values measured. For the large delamination defects (DL1), the lowest shallow delamination ER is 1377.30 kΩ-cm with a percent difference of 26.90%, the lowest percentage for point ⓕ. Similar observations were made for concrete near a rebar mesh and concrete with deep delamination, which has ER values of 821.67 kΩ-cm (% difference = 24.30) and 573.17 kΩ-cm (% difference = 47.13), the lowest in both of their respective locations. 

The discussion above validates the conclusions of existing research that ER values measured directly on top of a rebar will be relatively lower [37,50,52]. This also validates conclusions from previous papers that, in certain situations in which steel reinforcements are unavoidable during ER measurements, the probe should be placed perpendicular to the rebar to minimize the effect of the presence of rebar [54], as shown in configuration C4 in Figure 6. It can be pointed out in Table 1 that the ER values in C4, is within the range or higher than C1, C2, and C3, all of which were placed away from the location of the rebar. This also explains that measurements made at the test points ⓚ and ⓛ of the concrete slab specimen 2, located away from any rebar, result to overflowing (OF) values, which is higher than the values gathered at the shallow delamination defects (DL1) of the concrete slab specimen 1.

### 4.5. Effect of Surface Saturation

**Figure 11:**
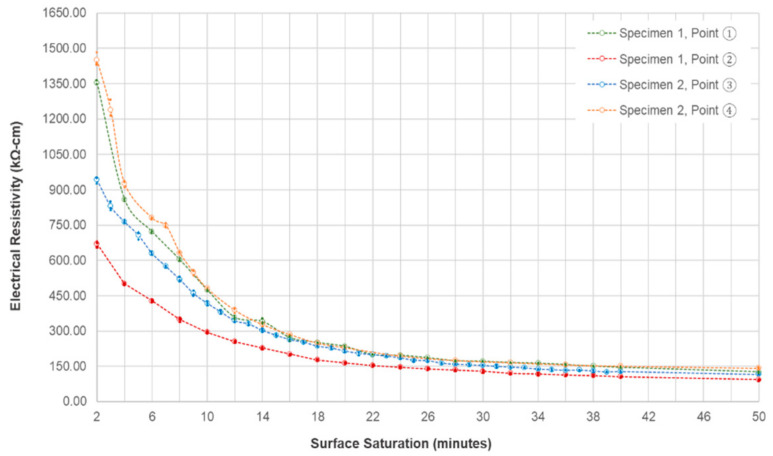
Effect of instantaneous saturation to the electrical resistivity measured with respect to time.

Figure 11 shows the effect of instantaneous saturation of the reinforced concrete slab surface to the measured ER, measured continuously for 50 min. It is observed that all the points considered in this test experienced decrease in ER. For the first two minutes of the saturation, the ER of points ① to ④ are 943.0 kΩ-cm, 1451.0 kΩ-cm, 1354.0 kΩ-cm, and 670.0 kΩ-cm respectively, which significantly decreased to 126.3 (point ①), 93.1 kΩ-cm (point ②), 115.0 kΩ-cm (point ③), and 142.0 kΩ-cm (point ④). It can be observed that there is a sudden decrease in the resistivity values of both points for the first 20 min of the saturations, while a gradual decrease was seen for the remaining minutes of saturating the concerned points of the specimen. This phenomenon can be explained by the study that the electrical resistivity has an inverse relationship with regards to the degree of saturation as established in prior research papers [49,57,58].

**Figure 12:**
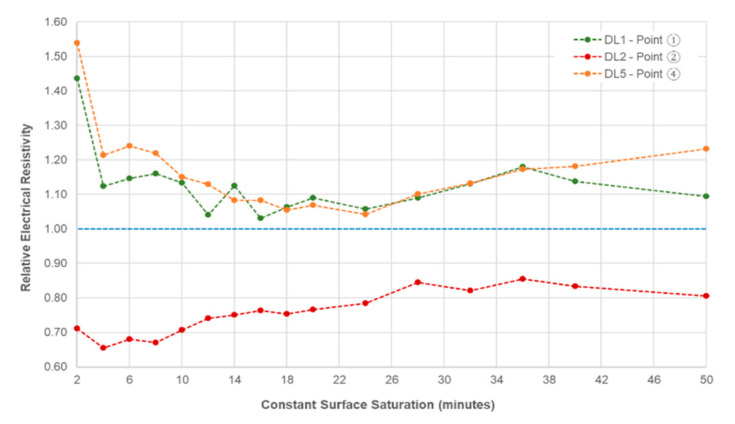
Relative electrical resistivity at delaminated zones with respect to continuous surface saturation in minutes.

In addition, the effect of delamination defects tends to decrease as the saturation time increases (or degree of saturation increases). Figure 12 shows the relative ER (ratio of the measured electrical resistivity at delaminated zones to the measured electrical resistivity at solid concrete) at delaminated zones with respect to continuous surface saturation in minutes. For shallow delamination defects, it can be observed that there is a large decrease in ER for the first four minutes of saturation, and a gradual fluctuating decrease for the next 20 min. For the shallow delamination defects, an increase in relative ER is observed, closing the gap at approximately 0.8, at the end of the saturation time. It should be noted that all the relative ER at delaminated zones only becomes nearer to 1.0 (reference for solid concrete resistivity), but gaps are still seen.

**Figure 13:**
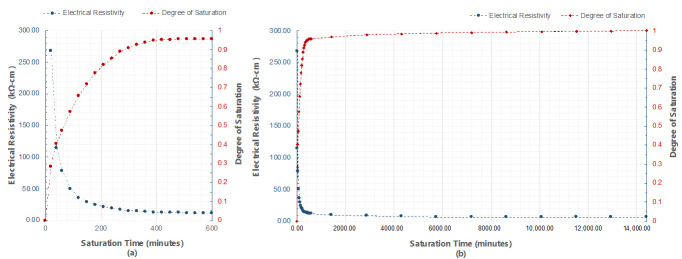
Relationship between electrical resistivity (ER) and degree of saturation with respect to saturation time of (**a**) 600 min, and (**b**) 14,400 min (10 days).

The effect of the surface saturation of concrete specimens to ER should be considered in practical engineering practice. AASHTO (American Association of State Highway and Transportation Office) [33] and a great deal of researchers [38,49,59] show that before determining the concrete resistivity, laboratory specimens should be fully saturated or in saturated surface dry condition. That is not the case for field measurements, because the saturation condition of concrete is exceedingly difficult to control. As shown in Figure 13, to reach a minimum value of 0.9 degrees of saturation, the concrete sample should be immersed for at least six hours, five days for a degree of saturation of 0.99, and seven days to be fully saturated. This figure also follows the trend of Figure 11 in the decline of the ER values. At the first 20 min of the saturation of the concrete specimens, the average electrical resistivity of the cylinder is 267.5 kΩ-cm, whereas the measured value for the concrete slab at the same time is 215.0 kΩ-cm. Concrete being a porous material, the degree of saturation depends mainly on its material composition and properties [49,60]. Since both the concrete slab and the cylinder have the same concrete mixture, the cylinder can be a good basis for identifying the saturation condition of the slab. It can be reasonably assumed that the degree of saturation of the slab after 20 and 40 min of saturation are approximately 0.28 and 0.40, respectively, based on the ER values on concrete cylinders at the same times. As mentioned above, it will take a couple of days before the concrete becomes fully saturated. This is impractical and not suggested in field measurements because NDTs are done at the day of actual inspection. It is suggested that before measuring the ER of concrete, the degree of saturation of the large structural elements such as slabs and deck should be the same to have a more accurate ER measurement. Having Figure 11 as reference, if engineers and maintenance personnel decide to saturate the specimens first before measurement, the saturation time of the concrete should be at least 20 to 30 min to significantly minimize the effect of the delamination defects. However, in some cases where ER measurements are done right away, either through a contact sponge attached at the electrodes of the Wenner probe device, through spraying of water on the surface, or by RABIT for bridges, it should be taken into consideration that the delamination defects will have a substantial effect on the concrete resistivity.

## 5. Conclusions

The effects of delamination defects to the electrical resistivity (ER) measurements were evaluated in this research paper. The researchers utilized the four-point (Wenner Probe) method in determining the resistivity of two reinforced concrete slab specimens. A numerical simulation through COMSOL Multiphysics was also conducted to be compared to the experimental data. In general, shallow delamination defects resulted in higher ER values, whereas probe configurations placed at deep delamination defects caused a decline in the measured concrete resistivity, all with reference to values gathered from solid concrete locations. In addition, the surface saturation of the concrete specimen decreases the values of electrical resistivities and minimizes the effect of delamination defects. The specific conclusions derived from this research are summarized as follows:

(1) ER measurements conducted in this paper are variably controlled and precise considering that the COV computed are lower or near the values set by AASHTO TP358-15. ER values gathered also follow a normal distribution curve, which means that the measured ER data in this study can be represented by two statistical parameters (i.e., mean value and standard deviation).
(2) The delamination defects in this study are divided into shallow delamination and deep delamination defects. Based on the experimental data gathered and the analysis of data conducted, it is deduced that shallow delamination defects (with depth of 50 mm), both small and large, contributes to a higher ER. On the other hand, small and large deep delamination defects (with depth of 250 mm) lead to a lower measured ER. The values gathered were compared to the ER of solid concrete. With reference to the average ER of solid concrete, the percent differences of shallow and deep delamination range from 26.90% to 49.35% and −17.19% to −47.19%, respectively.
(3) It is observed in this study that the size of the delamination also affects the value of ER. As the size of the shallow delamination defects increases, the measured ER increases. On the other hand, the size of deep delamination defects is inversely proportional with the measured ER. It should be taken into consideration that measurements are done with the presence of steel reinforcement that may influence the values of ER, specifically for deep delamination defects, which are located below two layers of rebars.
(4) It was concluded from the numerical simulation and experimental studies that ER values decrease with depth of delamination defects. For a plain concrete model, the relative ER closes to 1.0, whereas for reinforced concrete, relative ER decreases to 0.7. For the increase in size of delamination defects, shallow delamination defects in both plain and reinforced concrete models resulted in the increase in ER values. For deep delamination defects, the increase in size is negligible. Limitations are found in the numerical simulation, since other concrete properties and environmental factors such as degree of saturation, and the porous property of concrete were not included in the analysis. A more detailed and more systematic study of the effect of the presence of rebars, and/or the combination of the effect of the presence of reinforcements and delamination should be investigated both experimentally and numerically.
(5) The presence of steel reinforcement has an effect on the measurements, resulting in a lower value of ER. Among the probe configurations used, the configuration C5, where the device was placed directly on top of the rebar, produced the smallest ER, whereas measurements made using C4 (the probe is perpendicular to the rebar), and C6 (probe is placed diagonally to the mesh) were within the range of the first three configurations. It can be inferred in this study that the presence of rebar near locations of measurements leads to a lower resistivity value. Measurements done at DL5, in which rebars are farther as compared to the points considered in shallow delamination of the concrete slab specimen 1, have overflowing (OF), or very high measurements.
(6) It is established in this research that constant surface saturation decreases the value of ER. It is also established in this experiment that the gap of the ER measured along solid concrete locations and delaminated zones decreases due to the constant surface saturation of concrete. It is recommended in this study that in actual field measurements, to minimize the effect of delamination defects, saturation of the concrete surface should be done for at least 20–30 min. However, for measurements that are done right away (approximately at the first two minutes), delamination defects will greatly affect the ER measurements.

## Figures and Tables

**Table 1:** Average electrical resistivity of concrete slab specimens 1 and 2 at different probe configurations and different measurement points for large delamination defects.

Measured Location		Configuration											
		C1		C2		C3		C4		C5		C6	
		μ	COV	μ	COV	μ	COV	μ	COV	μ	COV	μ	COV
ⓐ	**Solid ^1^**	1021	6.06	1084	4.97	1023	4.36	-**^4^**		-		-	
ⓑ		1003	1.37	1086	1.48	1020	4.19						
ⓒ	**Solid ^2^**	1028	3.19	1123	5.26	929	5.69	1093	4.80	856	2.81	1009	4.46
ⓓ		1021	3.10	970	3.50	980	2.24	995	1.92	821	4.72	1054	3.30
ⓔ	**DL1**	1504	1.15	1474	2.16	1457	1.73	1439	2.19	1427	3.04	1471	1.49
ⓕ		1512	1.03	1449	2.64	1452	2.45	1477	3.40	1377	1.74	1414	1.95
ⓖ	**DL2**	663	3.80	699	6.69	678	5.59	656	7.08	573	4.48	635	6.47
ⓗ		646	6.41	676	2.48	651	3.67	708	2.54	588	6.01	686	5.12
ⓛ	**DL5**	OF **^3^**	-^5^	OF	-	OF	-	OF	-	OF	-	OF	-
ⓚ		OF	-	OF	-	OF	-	OF	-	OF	-	OF	-

Note: ^1^ over solid concrete and ^2^ over solid concrete near a reinforcing bar; ^3^ OF stands for “overflowing” and indicates that the measured ER value exceeds the range of ER measurement device. ^4^ Configurations C4-C6 not applicable to solid concrete. ^5^ Not applicable since no numerical data.

**Table 2:** Average electrical resistivity (ER) of concrete slab specimen 1 at different probe configurations and different measurement points for small delamination defects.

Measured Location		Configuration							
		C7		C8		C9		C10	
		μ	COV	μ	COV	μ	COV	μ	COV
ⓘ	**DL3**	1419	3.11	1362	3.35	1392	1.28	1384	5.64
ⓙ	**DL4**	838	2.72	733	3.76	615	5.94	746	5.31

**Table 3:** K-S test statistic (D) to check the normal distribution of the electrical resistivity measurement for large delamination of the concrete slab specimen 1.

Measured Location		Probe Configuration					
		C1	C2	C3	C4	C5	C6
ⓐ	**Solid ^1^**	0.0904	0.0983	0.0723	-	-	-
ⓑ		0.0885	0.1039	0.0731	-	-	-
ⓒ	**Solid ^2^**	0.1369	0.0793	0.1122	0.1499	0.0930	0.1530
ⓓ		0.1511	0.1072	0.1457	0.1087	0.0630	0.0664
ⓔ	**DL1**	0.1276	0.1182	0.0839	0.0814	0.1248	0.0737
ⓕ		0.0891	0.0735	0.0000	0.1010	0.1319	0.1345
ⓖ	**DL2**	0.1275	0.0923	0.1886	0.0804	0.1209	0.0934
ⓗ		0.0835	0.1073	0.1147	0.0779	0.2785	0.0766

Note: ^1^ over solid concrete and ^2^ over solid concrete near a reinforcing bar.

**Table 4:** K-S test statistic (D) to check the normal distribution of the electrical resistivity measurement for the small delamination defects of the concrete slab specimen 1.

Measurement Points		Probe Configuration			
		C7	C8	C9	C10
ⓘ	**DL3**	0.0971	0.1006	0.1281	0.1811
ⓙ	**DL4**	0.0841	0.0813	0.1286	0.1173

**Table 5:** Electrical resistivity of concrete slab at different sizes of delamination defects.

Square Dimension of Delamination (mm)	Electrical Resistivity [kΩ-cm]	
	Shallow	Deep
150	1362.0	733.4
300	1504.4	663.6
600	OF	Not available
